# Integral Utilization of Red Seaweed for Bioactive Production

**DOI:** 10.3390/md17060314

**Published:** 2019-05-28

**Authors:** Maria Dolores Torres, Noelia Flórez-Fernández, Herminia Domínguez

**Affiliations:** Department of Chemical Engineering, Faculty of Sciences, University of Vigo, Campus Ourense, As Lagoas, 32004 Ourense, Spain; matorres@uvigo.es (M.D.T.); noelia.florez@uvigo.es (N.F.-F.)

**Keywords:** red seaweed, bioactives, extraction, biorefinery

## Abstract

The hydrocolloids carrageenan and agar are the major fraction industrially extracted and commercialized from red seaweeds. However, this type of macroalgae also contains a variety of components with nutritional, functional and biological properties. In the context of sustainability and bioeconomy, where the integral utilization of the natural resources is incentivized, the sequential separation and valorization of seaweed components with biological properties of interest for food, nutraceuticals, cosmeceuticals and pharmaceuticals is proposed. In this work, a review of the available conventional and alternative greener and efficient extraction for obtaining red seaweed bioactives is presented. The potential of emerging technologies for the production of valuable oligomers from carrageenan and agar is also commented, and finally, the sequential extraction of the constituent fractions is discussed.

## 1. Introduction

Seaweeds are widespread and traditionally used in Eastern countries for food and for medicinal purposes. In Western countries, despite being included recently in the diet, direct human consumption is still unusual, mainly being used for the production of hydrocolloids with thickening and gelling properties. Among seaweeds, red algae (Rhodophyta) contain high amounts of polysaccharides (floridean starch and sulfated galactans, such as carrageenans or agarans), proteins and derived peptides (phycobiliproteins, phycolectins and mycosporine-like amino acids), minerals and other valuable compounds, such as polyphenols and lipids [1,2]. The whole algae of red seaweeds have been traditionally used as food, while agars and carrageenans have been extracted for multiple purposes, namely for food, pharmaceutical applications and biotechnological applications.

The reader can find compilations on the chemical and nutritional characteristics of seaweeds as a feed livestock resource [3] and its health-promoting properties [4], including the anticancer [5] and antiviral [6] features. Additionally, there have been some interesting reviews on the use of red seaweeds for carrageenans [7], agar and carrageenan oligosaccharides [8]. The extraction technology, which influences the composition, structure and properties of the target solutes, is conventionally addressed using chemicals, in long term operation, and with high energy consumption [9]. The recent developments of blue biotechnology and novel extraction techniques meeting the requirements of low cost, sustainability, food-compatibility and industrial scale feasibility to obtain seaweed components [10], the systematical selection of operational variables of emerging extraction technologies [11] or the use of alternative solvents, such as ionic liquids [12], or supercritical CO_2_ for macro and microalgae [13] have been reviewed. Scarcer studies are focused on the extraction of red seaweed components, such as carrageenan and agar fractions [9,14], photoprotective substances [15] and pigments [16]. Particular interest has been on the influence on the depolymerization of saccharidic and protein components, since both the type of extraction process and the operational conditions have to be controlled depending on the future uses [17]; however, further development and additional studies are still needed [18]. The mentioned studies are oriented to the selective extraction of some valuable fractions, but scarce information is found for the simultaneous utilization of the different components in a more rational scheme, following the philosophy of the biorefineries [19]. Biomass refineries, with a production scheme analogous to the petroleum refineries, are aimed at obtaining a wide range of products from renewable raw materials, including value added components for the food, cosmetic and pharmaceutical industries, as well as biofuels. These multistage multiproduct processes are based on the sequential fractionation of the biomass and on their subsequent physical, chemical or biotechnological transformation into the target final products. This sustainable approach adopts an integral utilization of resources, promoting the development of a marine bio-economy.

The present review presents an overview of the properties and potential applications of red seaweed bioactives, the specific technologies for extraction and also for the depolymerization of agar and carrageenan into oligosaccharides, as well as the potential of these techniques for the extraction of other red seaweed components. Both conventional and emerging extraction and depolymerization technologies are discussed with the aim of promoting the sustainability based on (i) the development of clean processes and (ii) the integral utilization and valorization of resources following the philosophy of biomass biorefineries.

## 2. Components: Properties and Extraction

### 2.1. Polysaccharides

Polysaccharides are the main components in marine algae according to their abundance and their current commercial value based on their technological features [4,20]. More recently, attention has been directed to their health benefits [21,22]. These polysaccharides, generally not digested by humans, are considered to be dietary fibers [23]. The composition, structure and rheological properties are influenced by the algal source, life-stage, growth, environment and by the extraction method [24]. Agars and carrageenans are major cell wall polysaccharides in red macroalgae, also known as galactans, accounting for up to 40–50% of the dry weight. They are highly anionic homopolysaccharides, composed of a backbone built from disaccharide blocks of d-galactose and 3,6-anhydrogalactose (l-AHG in agar and d-AHG in carrageenan) with different sulfation, methylation and pyruvation patterns that vary among species [21,25]. The high electronegative charge density from their sulfated esters favors the electrostatic interactions with specific proteins, determining their biological effects, which are also closely related to the structural features [20,26,27,28]. Proteins, minerals and lipids also confer red seaweed important structural value [29].

#### 2.1.1. Agar

- Composition, structure, occurrence and properties

**Figure 1:**
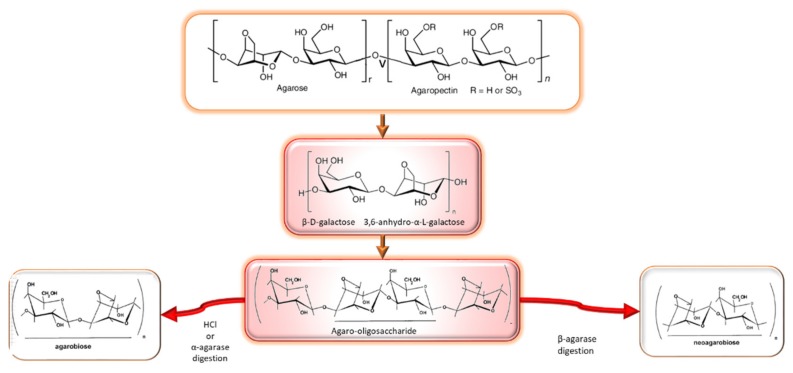
Scheme of the agar constituents (agarose and agaropectine) and different derived molecules with biological activities, adapted from [43,44].

Agar is a linear polysaccharide composed of alternating (1,3) linked d-galactose and (1,4) linked 3,6-anhydro-l-galactose [25] and substituted in some degree by sulfate, methyl or pyruvate groups [30,31,32]. The molecular structure of agar polysaccharides, particularly the type and location of sulfate esters, appears to be species-specific [33]. Agar has two different constituents: agarose and agaropectin (Figure 1). Agarose is a neutral linear polysaccharide composed of three linked β-d-galactose and four linked 3,6-anhydro-α-l-galactose. Agaropectin is an acid polysaccharide containing sulfate groups, pyruvic acid, and d-glucuronic acid conjugated to agarobiose. Agarose accounts for up to 70% of the mixture and is responsible for gelling, whereas agaropectin is responsible for thickening characteristics. Different derived agarose molecules can be obtained from chemical or enzymatic degradation. Most of the corresponding hydrolysis products such as agarooligosaccharides (AOSs), neoagarooligosaccharides (NAOSs), neoagarobiose (NAB) and 3,6-anhydro-l-galactose (l-AHG) exhibit biological activities [34].

Agar is mainly found in the cell matrix of seaweeds of the order Gelidiales (*Gelidium* and *Pterocladia*) and Gracilariales (*Gracilaria* and *Hydropuntia*), which have become the major worldwide source. Its abundance and easier exploitation made *G. tenuistipitata* an economically important raw material for agar production [35]. In comparison with agars from *Gelidium* and *Pterocladia*, agars from *Gracilaria* can have higher degrees of sulfation, methoxylation and pyruvylation [31].

The agar properties are dependent on the species and environmental characteristics of the collection or cultivation area, such as season, life cycle and geographical features [36,37] and the storage, extraction processes and postharvest storage [24,32,38,39,40,41]. The quality of agar is determined by the type, pattern and degree of substitution as well as molecular weight, chemical composition (pyruvate, methyoxyl and sulfate) and physical properties (gel strength, gel syneresis, viscosity, gelling and melting temperatures) that determine its market value [30,32,42]. The agar gel strength, in terms of elastic modulus (G′), of systems formulated at 1.5% agar in milli-Q water is around 238 Pa at 25 °C, with gelling temperatures of 48 °C, and those agars with gel strengths greater than 6.9 × 10^4^ Pa are referred to as high quality agars [24].

Agar is a generally recognized as safe (GRAS) food additive in the United States and a food additive approved in Europe (E406). Agar cannot be digested in the gastrointestinal tract because humans lack α/β-agarases, but can be metabolized by intestinal bacteria to d-galactose [45]. Agar is demanded as gelling agent and stabilizing agent, and as cryoprotectants in the pharmaceutical, cosmetics and food industries [39,46,47,48,49]. The human food industry demands for 80% production, and biotechnological applications for the remaining 20% [50]. The importance of these products is based on high market demand for agar and the higher price compared to alginates and carrageenans [24,32,51]. It is used as a gelling, thickening and stabilizing agent in food formulations and it has also been used in microbiological media and in chromatographic techniques. Most native agars from *Gracilaria* are not bacteriological grade agar due to their high content of methoxyls, but they can be food and reactive grade [52].

- Extraction processes: conventional and emerging technologies

The storage conditions and duration before extraction affects the agar quality from *Gracilaria*, since seaweeds are susceptible to degradation by agarolytic enzymes and bacteria. Some species from temperate and cold water could be more resistant to hydrolysis during storage. Postharvest treatment with acid, alkali or formaldehyde is necessary to prevent enzymatic and microbial degradation [32,37,40,47,50,53]. Another factor requiring attention after harvesting algae is correct drying under 20% moisture and packing, and avoid wetting during the transporting and storage period, but dewatering pre-treatments have to be defined according to the species and to collection season [54].

Although *Gelidium* agar has better quality and is easily extracted with boiling water, the gelling ability of agars from *Gracilaria sp* can be enhanced by an alkali pretreatment to convert α-l-galactose 6 sulfate into 3,6-anhydro-α-l-galactose. This treatment reduces the sulfate content and improves the gelling properties as evidenced by higher gel strength, gelling, melting temperatures and viscosity [42]. Generally, the alkali-treatment was most effective for obtaining more galactose-rich hydrocolloids [24]. However, agar degradation and diffusion towards the aqueous medium could occur, reducing the extraction yield [29,55], although in some cases, no reduction was observed [24]. Alkaline pretreatment variables, such as alkaly type and concentration or heating time and temperature affected the yield and quality of the agar. Regardless the alkaline concentration, NaOH rendered agar with a higher quality than KOH [35,56]. Compilations of conditions are also found in [37], being the optimal in the range 5–7% NaOH, up to 80–100 °C for 0.5–3 h [29,35,46,48,50], but higher alkali concentrations (10%) [55], shorter times [57] and the application of several stages [53] have also been reported. An alternative pretreatment was proposed by Roleda [58], which consisted of soaking the *Gelidiella acerosa* air dried sample in 0.5% acetic acid for 1 h at 16–20 °C, then 1 h steam pressure at 15–20 psi and boiling at 100 °C. Freile-Pelegrín [59] proposed the cultivation of *Gracilaria cornes* under dark and salinity treatments (50 and 25% salinity) to replace the alkali treatment. Pigments, such as chlorophyll, carotenoids and phycoerythrobilin, can be leached out during the alkaline pretreatment and an alternative environmentally friendly scalable photobleaching process for *Gracilaria asiatica* and *Gracilaria lemaneiformis* with 3–5% NaOH and photobleaching for 5 h was proposed [60]. The pigments and the agar sulfate contents decreased during the photobleaching agar extraction process, and the gel strength increased during the photolysis.

The industrial agar extraction process is based on using hot water during several hours under conventional heating, a time-consuming process requiring high solvent consumption and generating large amounts of waste disposal. Therefore, water recycling has been suggested [36]. Compilations on agar yield (10–43.4%) can be found in [37]. Cold extraction with distilled water at room temperature was reported for *Gracilaria birdiae* [61], but agar extracts prepared at 20 °C showed a wider size distribution (1–30,000 kDa) [62]. For the chemical liquefaction of agarose, acid prehydrolysis has been commonly employed [63,64,65]. Mild conditions, such as low acid concentrations, low temperatures or short reaction times, result in even-numbered oligosaccharides due to the preferential cleavage of α-1,3-glycosidic linkages and the release of the acid-labile l-AHG at the reducing end converts even-numbered AOSs into odd-numbered ones. The released l-AHG is readily degraded into 5-hydroxymethylfurfural [65]. Table 1 summarizes some representative examples of the conventional and emerging technologies proposed for agar extraction. Another disadvantage of alkali treatment is the generation of effluents with environmental impact if not properly treated [66]. Enzymatic treatment could be a more ecofriendly alternative to improve the gel strength of agar. Shukla [67] proposed the use of sulfatase/sulfohydrolase to decrease the sulfate content and increase both the 3,6-anhydrogalactose content and gel strength of agar. However, the cost could not make the process commercially competitive with this sulfatase/sulfohydrolase compared to alkaline treatment [22].

Processes based on combined heat and ultrasound treatments would enable reducing the amount of time and energy needed. Martínez-Sanz et al., [29] observed a four-fold reduction in time without affecting the yields and properties of *Gelidium sesquipedale* agar-based extracts. The extracts also contained proteins, polyphenols and minerals, conferring antioxidant capacity to the browned softer gels. In contrast, an alkali pre-treatment could yield almost pure agars with higher molecular weights and crystallinities and resulted in stiffer gels, but lower extraction yields.

Microwave assisted extraction (MAE) allowed reducing the required 2–4 h for agar extraction in conventional processes to a very short period, consuming less energy and solvent volume and reducing waste disposal requirements [9,48]. Navarro and Stortz [68] used microwave assisted alkaline modification to improve the gelling properties of carrageenans from *Iridaea undulosa* and porphyran from *Porphyra columbina*. Substantial depolymerization of *Gracilaria vermiculophylla* agar was observed in microwave assisted extraction with lower values of viscosity and molecular weight (54 kDa against 111 kDa) and methylation degree than those obtained in conventional extraction [36]. Intermittent microwave treatment was proposed for the extraction of sulfated porphyran from *Porphyra dentata* with ethanol and the gelling capacity of extracted porphyran was not affected [69].

Other technologies have been proposed, such as ionic liquid-based extraction [12], radiation to increase the agar yield from *Gelidiella acerosa*, at 15 kGy yield increased, but the gel strength decreased and the sulfate level did not vary significantly [70].

**Table 1:** Some examples of technologies proposed for agar extraction.

Pretreatment/Extraction	Seaweed	Gel Properties	Reference
P: - E: Distilled water; pH 6.3–6.4; 100 °C, 1.5 h; ethanol precipitation	*Gracilaria cornes*	GS: (1.2–2.5) × 10^4^; Tg: 39.2–41.8; Tm: 74.3–82.6; Mw: ND	[59]
P: 1–15% NaOH, 90 °C, 1 h, 0.025% HCl, 1 h E: Water, 100 °C, 2 h, ethanol precipitation	*Gracilaria verrucosa*	GS: (1.6–1.8, 2.6–2.7) × 10^4^; Tg: 32–43; Tm: 49–80.5; Mw: ND	[55]
P: - E: Distilled water, 20–28 °C, 15 h, ethanol precipitation	*Gracilaria birdiae*	GS: ND; Tg: ND; Tm: ND; Mw: 1–30,000	[61]
P: - E: Water, 80–100 °C, 2–4 h; ethanol precipitation	*Hydropuntia cornea*	GS: (0.7–1.3) × 10^4^; Tg: 25–32.1; Tm: 65–79; Mw: 342–371 kDa	[71]
^1^ P: 5–7% NaOH, 80–100 °C, 0.5–3 h E: Water, 80 °C, pH 6.2, 90 min, ethanol precipitation	*Gracilaria vermiculophylla*	GS: (0.9–1.2) × 10^5^; Tg: 52–68; Tm: 92–95; Mw: ND	[37]
^1^ P: 1–5% NaOH, 30–85 °C, 1–2 h E: Water, 700–115 °C, 2–3 h, 1–2 stages, ethanol precipitation	*Gracilaria corticata*, *Gracilaria eucheumoides*, *Gracilaria cliftonii*, *Gracilaria lemaneiformis*	GS: (1.2–4.2) × 10^4^; Tg: ~32; Tm: ~78; Mw: ND	[50,53,72,73]
P: 5% NaOH, 1–48 h, room temperature. Dil. H_2_SO_4_, 15 min E: Water, 100 °C, 1 h 30 min, ethanol precipitation	*Gracilaria manilaensis*	GS: (1–4.9) × 10^4^; Tg: ND; Tm: ND; Mw: ND	[56]
P: - E: Pressurized water extraction, 120 °C, 15 min, ethanol precipitation	*Gracilaria vermiculophylla*	GS: 1.3 × 10^5^; Tg: 40.7; Tm: 93.1; Mw: ND	[48]
P: Acetic acid, 16–20 °C, 1 h E: Steam pressure, 15–20 psi; ethanol precipitation	*Gelidiella acerosa*	GS: (4.9–6.9) × 10^4^; Tg: 42–47; Tm: 90–98; Mw: ND	[58,74]
^1^ P: 2.5 M NaOH, 90 °C, 2 h E: Water, 90 °C, 2 h; ultrasound assisted, 30 min, 400 w, 24 kHz; ethanol precipitation	*Gelidium sesquipedale*	GS: (0.2–1.2) × 10^5^; Tg: ND; Tm: ND; Mw: (2.5–11) × 10^5^	[29]
^1^ P: 0.1 M NaOH, 22 °C E: Enzyme (60 °C, 12 h, pH 8) and ultrasound assisted extraction (60 °C, 30 min, 60 W); ethanol precipitation	*Gracilaria birdiae*	GS: ND; Tg: ND; Tm: ND; Mw: 20–45	[75]
P: - E: Protease digestion, 60 °C, 6 h, pH 5	*Gracilaria cornea*	GS: ND; Tg: ND; Tm: ND; Mw: ND	[76]
P: Radiation, at 5–15 kGy E: Water, 95–100 °C or pressure cooking 121 °C, 15 psi, 1 h; ethanol precipitation	*Gelidiella acerosa*	GS: (2.5–6.0) × 10^4^; Tg: ND; Tm: ND; Mw: ND	[70]

^1^ Optional pretreatment; P: pretreatment conditions; E: extraction conditions; GS: gel strength (G′, elastic modulus at 25 °C, Pa); Tg: gelling temperature (°C); Tm: Melting temperature (°C); MW: Molecular weight (kDa); ND: not determined.

- Agarooligosaccharides: properties and production strategies

Two oligosaccharides can be formed depending on the moiety of end sugar, namely, agaro-oligosaccharides and neoagaro-oligosaccharides [77]. Neoagarobiose, α-l-3,6-anhydro-l-galactosyl-(1→3)-β-d-galactopyranose, is the basic unit of neoagarooligosaccharides. Neoagarooligosaccha-rides were found to be safe up to 5000 mg/kg body weight in acute oral toxicity tests with rat and beagle dog models [45].

**Figure 2:**
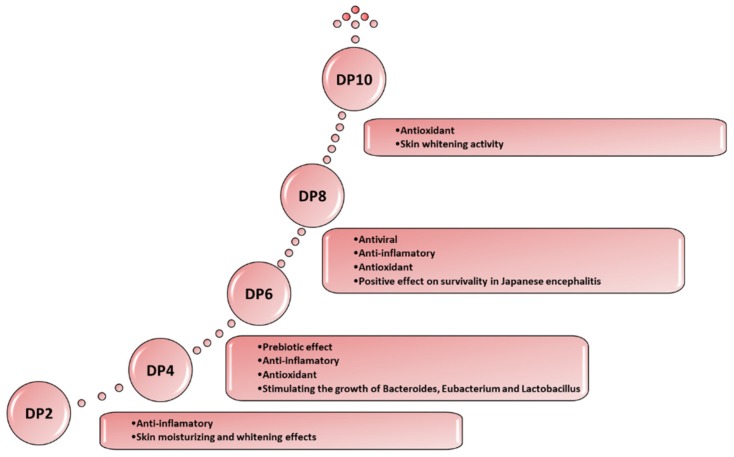
Influence of the depolymerization degree (DP) of agar oligomers on their biological properties [34,77,84,87,92,93].

The biological activities of agar oligosaccharides include anti-microbial, antiviral [78], prebiotic [79], anti-tumoral, immunomodulatory, anti-inflammatory [76,80,81,82,83,84,85,86,87], glucosidase inhibitory [77], anticariogenic [34], hepatoprotective [83], antioxidant [77,83] and other properties of interest for skin care [45,77,84,85] (Figure 2). Liu et al. [86] summarized research progress on biological activities of agaro-oligosaccharide. Agaro-oligosaccharides display antioxidant effects which differ according to their degree of polymerization [77]; additionally, Kazłowski et al. [87] summarized the influence of the degree of polymerization (DP) of agar oligomers on their physiological activities. Agarose is biocompatible and has been used for neural and cartilage tissue repair [88] and for the preparation of biomaterials [89,90]. Due to its low cell adhesiveness and slow degradation rate, agarose was composited with fast degradable biomaterials for drug delivery, tissue engineering and wound healing [91].

Agaro-oligosaccharides (AOS) are conventionally prepared by acid hydrolysis of agars; however, this method produces substantial pollution and wastes. Alternative strategies have also been proposed using the same subsequent stages for purification, usually based on ultrafiltration, ethanolic precipitation, purification by chromatography and further in activated carbon [77,83,87].

Several acids have been used to hydrolyze agar. Chen [77] compared the use of hydrochloric acid, citric acid and cationic exchange resin; the latter avoided the neutralization step and offered higher yield of agaro-oligosaccharides with high DP (octaose and decaose) and low content of agarobiose. Hydrochloric acid hydrolysis produced DP lower than 6, whereas citric acid yielded small amount of oligosaccharides, mainly agarooctaose and agarodecaose.

Alternative methods have been used to hydrolyze agar, such as enzymatic, physical and chemical degradation. Enzymatic hydrolysis, which can be performed by agarases [77], show disadvantages such as the low activity, low stability and productivity, which limit their wide application in industry. However, chemical degradation, especially acid hydrolysis, is available for industrial preparation because of its simplicity, rapidity, low cost and high yield [77]. Different bacteria have been used as a source of agarolytic enzymes, i.e., *Flammeovirga pacifica* [83], *Streptomyces coelicolor* [45] or *Agarivorans* sp. JA-1 [84]. Agar oligosaccharides can be produced by hydrolysis using chemicals or agarolytic enzymes. Since agarose comprises alternating l-AHG and d-galactose units linked by α-1,3- and β-1,4-glycosidic bonds, two types of agarases exist: α-agarases cleave the α-1,3 linkages of agarose endolytically and produce agaro-oligosaccharides AOSs as the reaction products. Neoagarooligosaccharides are prepared from agar by β-agarase hydrolysis, by cleaving the β-1,4-glycosidic linkages of agarose endolytically or exolytically, and also releases neoagarooligosaccharides with neoagarobiose or neogarobiose alone, respectively. Agaro-oligosaccharides obtained by enzymatic degradation exhibited high solubility percentages, water and oil absorption capacities, as well as considerable 2,2-diphenyl-1-picrylhydrazyl (DPPH) and 2,2′-azino-bis(3-ethylbenzothiazoline-6-sulphonic acid (ABTS^+^) radical scavenging and ferric reducing antioxidant activities, depending on the degree hydrolysis [94]. Kazłowski et al. [44,87] prepared neoagaro-oligosaccharides by β-agarase digestion and agaro-oligosaccharides by HCl hydrolysis from agarose and observed that the enzymatically prepared oligosaccharides usually show a low DP and a broad range of bioactivities, whereas those from acid hydrolysis contain only oligosaccharides with odd numbers of sugar unit [83].

The agaro-oligosaccharides, obtained from commercial agarose through enzymatic hydrolysis, did not show improvement on its oil and water absorption capacities. Furthermore, a higher degree hydrolysis could lead to increase the reducing capacity and antiradical properties (Table 2).

Zou et al. [93] prepared low molecular weight polysaccharides (3.2, 10.5, 29.0, and 48.8 kDa) from *Pyropia yezoensis* using microwave-assisted acid hydrolysis. The lower molecular weight (Mw) product (3.2 kDa) was the most efficient protecting wheat seedlings against salt stress. These authors also indicated that microwave irradiation accelerated the reaction rate. Stronger gels were also obtained using microwave assisted extraction when compared with gels produced applying the traditional method. The average sulfate content was similar to the obtained by the traditional method from *G. vermiculophylla* produced in the selected integrated multitrophic aquaculture (IMTA) system. However, radical depolymerization assisted by ultrasounds induced a loss of sulfate functions in addition to the shortening of the polysaccharides chain length [97]. It should be indicated that the microwave assisted extraction approach requires less energy and solvent than conventional processes, while generating fewer wastes.

Free-radical depolymerization of polysaccharides, based on the formation of free radicals ·OH by the Fenton reaction using a metallic catalyst, was proposed as a reproducible scalable technique for the degradation of polysaccharides without changes in structural features, producing an average molar mass of 1500 kDa [95].

High-pressure homogenization performed on the polysaccharide of *Halymenia durvillei* showed their feasibility and effectiveness and showed that an advantage of the degradation at high pressure was the ease and speed of the preparation [95].

It has been suggested that ultrasound promotes the extraction of other non-sulfated polysaccharides [75]. One of the fractions obtained by ultrasonic degradation of *Porphyra yezoensis* polysaccharides did not change the main structure of polysaccharides and enhanced the antioxidant properties of the agar fractions [96].

Combination of techniques was also useful, enzyme and ultrasound assisted extraction led to the same sulfated polysaccharides from *Gracilaria birdiae* [75], but the yield was higher when both techniques were jointly applied to alkaline treated seaweeds. Combined techniques were used in the extraction of pigments and sulfated polysaccharides from the red alga *G. verrucosa*. The method is easy to use, allows the extraction of pigments and agar highly quantitative in one step. Sulfated polysaccharides obtained were similar to agar extracted directly from dried material without any treatment. Compared to the common agar extraction method, enzyme mixtures tested for R-phycoerythrin can be proposed as pretreatment for agar extraction. However, Öğretmen and Duyar [98] observed that autoclave provided lower agar yields than water bath from Gelidium latifolium. 

Agaro-oligosaccharide dried products show high thermal and pH stability; however, the drying step is highly relevant to other product properties, both functional (water solubility index, water absorption capacity and oil absorption capacity) and antioxidant (radical scavenger and reducing capacity). Kang et al. [94] observed the highest solubility index in spray dried products and the highest water and oil absorption capacities in freeze dried products, which showed minimal color deterioration and higher oxidative stability, whereas the oven drying could be more deleterious. Antiradical properties were high in freeze-dried and spray-dried oligosaccharide powders.

#### 2.1.2. Carrageenan

- Composition, structure, occurrence and properties

**Figure 3:**
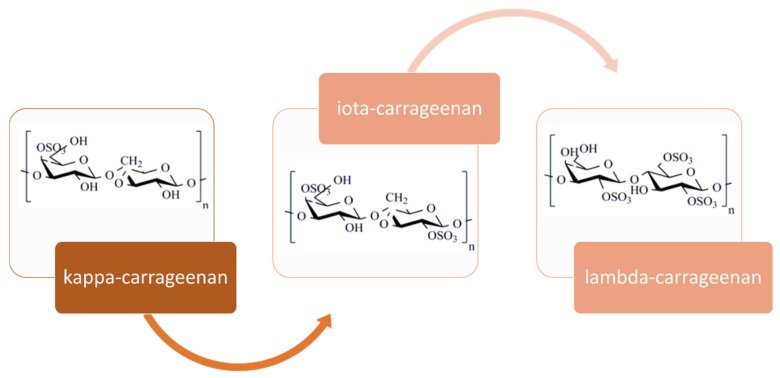
Repeating units in the main three types of carrageenan with commercial interest, adapted from Pereira [99].

Carrageenans are high-molecular-weight linear hydrophilic, sulfated galactans formed by alternate units of d-galactose and 3,6-anhydrogalactose alternately linked by α-1,3 and β-1,4 glycosidic linkages. They can be classified according to differences in their average molecular mass and to the number and the position of the sulfate ester groups and the occurrence of a 3,6 anhydro-ring in the α-linked galactose. The three types with higher commercial importance, namely κ-, ι- and λ-carrageenan, are presented in Figure 3.

Carrageenans are mainly obtained from the genus *Chondrus*, *Eucheuma*, *Gigartina*, *Iridae*, *Furcellaria* and *Hypnea*, and the expansion and increased demand led to the introduction of the cultivation of *Kappaphycus alvarezii* and *Eucheuma denticulatum*, with a predominant content of k- and i-carrageenan, respectively, available all year [100]. *Chondrus crispus*, the original source, which contains a mixture of k- and λ- carrageenan, could be a model organism [101] and there is a renewed interest in the cultivation for cold water carrageenophytes, although the economics need to be carefully considered [102]. The differences in composition and molecular conformation determines the rheological profiles, gel properties and textures of carrageenans as well as the interactions with other gelling agents and food ingredients [103].

The three most commercially exploited carrageenans are kappa (κ-), iota (ι-) and lambda (λ-) carrageenans, which can be separately provided or as a well-defined mixture, since most of the seaweeds contain hybrid carrageenans [22]. Mu and nu carrageenans are the precursors in carrageenan, which are converted to kappa and iota, respectively, by means of alkaline modification. In the natural state, unmodified kappa and iota carrageenans account for around 30% mu and nu-carrageenans, remaining less than 5% after alkaline modification, randomly distributed within the repeating structures [103]. Origin, species or extraction processing conditions notably affect the type and quality of carrageenan isolated. The carrageenan types vary among species from the *Cystocloniaceae* family predominantly produce iota-carrageenans; the *Gigartinaceae* family lead to hybrid kappa-iota carrageenans and lambda-family carrageenans (sporophytic plants); the *Phyllophoraceae* family produces kappa-iota hybrid-carrageenans [104]. Commonly, κ-carrageenan is commercially isolated from *Kappaphycus alvarezii* red seaweed through a hot extraction process, whereas λ-carrageenan is more hygroscopic and it is usually extracted from red seaweeds of the genera *Gigartina* or *Chondrus* by drum dryer or alcohol precipitation process [105]. Concerning ι-carrageenan, it is commercially extracted from *Eucheuma denticulatum* by the freeze thaw or gel process [106]. The highest carrageenan yields can exceed 70% (dry basis) for some species such as *B. gelatinum*, *K. alvarezii* or *K. striatum*. Other species have values close to 30%, such as *E. denticulatum* or *C. crispus*. Sulfate content in carrageenans varies from 20% in κ-carrageenan, to 33% in ι-carrageenan and to 41% in λ-carrageenan [107]. It is well known that the carrageenans gelation process is affected by the biopolymer content, temperature, ionic strength of the solution and the cation type and content, being the most effective K^+^ and Rb^+^. In general, iota carrageenans form soft and elastic gels with higher gelling temperatures than κ-carrageenans [108]. In contrast, λ-carrageenan did not form gels, being used solely as thickening agent [109]. Gelling temperatures ranged from 32 to 36 °C for the κ-carrageenans, whereas ι-carrageenans exhibited values of 70–74 °C. Gel strengths, elastic modulus at 25 °C, varied between 4000–6500 Pa for alkali pre-treated samples [24].

Carrageenan is a natural ingredient, used for decades in food applications and generally recognized as safe (GRAS) by the Food and Drug Administration; furthermore, carrageenan and semi-refined carrageenan are food additives (E-407 and E407a, respectively) approved by European Food Safety Authority. The food viscosity specification is equivalent to an average molecular weight greater than 100 kDa, and commercial food carrageenan have Mw in the range 200–800 kDa [102,103]. Carrageenan is not degraded nor absorbed in the gastrointestinal tract [110,111,112] and should not contain low Mw fractions, poligeenan or degraded carrageenan, since it exhibits toxicological properties at high doses [110] and can induce gastrointestinal irritation and cancer in animal models [112] or can lead to cell death and inflammatory responses on human colonic epithelial cells [113,114], bacterial dysbiosis and shifted community composition [115] and decreased anti-inflammatory bacteria [116]. Further studies of its impact on digestive proteolysis, the colon microbiome and inflammation [117,118,119] and the effects on predisposed populations [119,120] are required. Carrageenans are also used for the pharmaceutical sector [106,107], based on anti-inflammatory [121], antiviral [6,122,123,124,125,126], anticoagulant [17,127], immunomodulatory [128], antitumoral [129], antioxidant [17,129,130], anti-angiogenic [97] and neuroprotective [129] activities. The biological properties of carrageenans have been reviewed in several publications [28,119,131,132,133].

Carrageenans are widely used as an inflammatory inducing agent in experimental animals [134], and have also been proposed for the immobilization and encapsulation of biocatalysts [135], for entrapping lactic bacteria and enzymes [136], for microencapsulation of probiotics in mixtures of k-carrageenan with carboxymethylcellulose [137]. Recent pharmaceutical applications are found in drug delivery and for tissue regeneration [138]. Carrageenans are highly biocompatible and are used as ingredients for films, beads, microparticles, nanoparticles, hydrogels, inhalable and injectable systems [107,108,139] used alone or in combination with other polymers, and its mucoadhesive properties have been exploited in the preparation of aerogel microparticles for mucosal drug delivery [140].

The role of carrageenans in agriculture has also been confirmed [141,142,143,144,145,146,147,148], since it stimulates growth [149] and increases defense responses against viruses [141,148] and abiotic stresses [93].

- Extraction processes: conventional and emerging technologies

Specific details of the commercial extraction processes are trade secrets for the manufacturers of carrageenan. The seaweed is usually dried quickly to prevent degradation during transportation to the processing facilities. The original method to produce the commercial carrageenans is based on washing to remove impurities, such as sand, epiphytes and salt and the carrageenan extraction in a hot aqueous solution, neutral or alkaline, filtration, recovery from the solution by alcohol precipitation, separation of the precipitate, drying and milling. However, this method is time and energy consuming, and has a low extraction efficiency. Alternatively, extraction of minerals, protein and lipids can be proposed, leaving in the raffinate the carrageenan and cellulose as a semi-refined low purity carrageenan. The main variables during extraction, namely temperature, pH, time and alkaline pre-treatment (alkaline agent, concentration and time), have to be optimized for each seaweed to maximize the structural and gelling properties [150,151]. The alkali pretreatment of red seaweed increases the ratio of ι- versus κ-hybrid [17], but usually, an excessive alkali content and prolonged treatment time can lower molecular mass and depressed gel properties [150]. Table 3 shows some representative examples of carrageenan extraction procedures.

Alternatively, low temperature has been proposed to extract low-molecular-weight carrageenans [155] and to maintain reducing, antiradical and anticoagulant activities, probably due to the higher sulfate content, which would be lost after hot-water, acid and alkali treatments [17]. Other options can lower time, energy demand and the consumption of water, chemicals and solvents. Among the novel extraction techniques to enhance the extraction efficiency are pressurized solvent extraction, microwave-, ultrasonic- and enzyme-assisted extractions [7,9,14].

Microwave-assisted extraction offers a reduction in time and energy consumption, thus enhancing the process efficiency [9]. Operating in closed vessel, more efficient desulfation was observed and the κ/ι hybrid carrageenan obtained was comparable to that extracted by the conventional technique [154]. Boulho et al. [152] did not observe significant differences in the carrageenan (predominantly iota-) yield from *Solieria chordalis*, were observed between MAE and conventional method under alkaline conditions, and the product showed antiviral activity against *Herpes simplex* virus type 1. Almutairi et al. [156] reported λ-carrageenan discoloration occurring during microwave irradiation for the aqueous solutions exposed to microwave heating. 

The ultrasound assisted processes both alkaline and aqueous, shortened extraction times compared to the conventional method, avoiding degradation of labile compounds, showing a slight variation in sulfate, AG and Gal contents and viscosity [153]. Youssof et al. [7] reported that they doubled the yields attained in four–eight longer times with conventional extraction without affecting the chemical structure and molar mass distribution of carrageenans.

- Carraoligosaccharides: properties and production strategies

Oligocarrageenans are oligomers of sulfated galactose, usually DP 2–20 [148,157], prepared by depolymerization by acid hydrolysis. Despite the degraded carrageenan caused significant mucosal ulceration of the colon, associated to histopathological changes, epithelial thinning, slight erosion, cellular infiltration and other negative changes in animal organisms [143], carrageenan oligosaccharides exhibit several biological activities, influenced by their molecular weight and sulfation degree.

Carrageenan oligosaccharides show scavenging properties against reactive oxygen species [158], hydroxyl radicals, superoxide anion, nitric oxide and hydrogen peroxide [159]. They also present anti-inflammatory and immunomodulatory [6,131,160,161], anticoagulant [158], antimicrobial and antiviral [6,148,158,162,163,164] and healing [165] properties. They also showed anticarcinogenic action [158,166,167,168,169], with low cytotoxicity [167] and synergistic effects with conventional drugs, improving the immunocompetence damaged by these drugs. The oligocarrageenans promote plant growth by enhancing photosynthesis, nitrogen assimilation, embryogenesis, basal metabolism, cell division, regulation of phytohormone synthesis [170,171,172,173] and by increasing protection against viral, fungal and bacterial infections [147,148,174,175], partly due to the accumulation of compounds with antimicrobial activity [148]. 

Partial depolymerization by chemical or enzymatic hydrolysis to obtain a range of oligosaccharides is a common strategy for structural analysis and for characterization of activity [176,177]. The biological profile of the products may be influenced by the depolymerization method, since it affects their size and molecular weight. In addition, carrageenan or their derived oligosaccharides may also be chemically modified by oversulfation, desulfation, acetylation or phosphorylation to achieve better physicochemical and biological properties [28,160,164,176], i.e., the antioxidant [176] and antiviral [178] activities.

Acid hydrolysis has been considered as a common and rapid method. The presence of acid and oxidizing agents may induce carrageenan depolymerization through cleavage of glycosidic linkages, a process accelerated by dissolved oxygen, high temperature and low pH. In order to limit undesirable degradation, high temperature and short time mild acid hydrolysis is preferred [164]. Karlsson and Singh [179] reported that carrageenans were stable to desulfation during acid (pH 2) hydrolysis at 35 and 55 °C. Kalitnik et al. [161] used mild acid hydrolysis of *Chondrus armatus* κ-carrageenan under conditions avoiding excess destruction of 3,6-AGal and observed that mild and acid hydrolysis cause breakage of inside α-1,3 links, producing mainly odd-numbered oligosaccharides. Mild acid hydrolysis at 37 ºC increased yields of even fractions in comparison with those obtained at 60 °C [180]. 

Enzymatic hydrolysis offers advantages due to its high efficiency under mild conditions, avoids the use of polluting chemicals, and the resulting oligosaccharides generated show higher homogeneity and lower polydispersity, thus providing compounds with improved and reproducible biological properties [107]. In addition, it avoids side reactions leading to undesired modifications of the native structure and the release of high amounts of monosaccharides and undesirable toxic products [8]. The enzymatic method, either using non-specific commercial enzymes or carragenases, is a relatively costly alternative. Carrageenases, produced only by marine gram negative bacterial species, are endohydrolases that hydrolyze the internal β-1,4 linkages in carrageenans [22,107,181]. Table 4 summarizes some representative examples of depolymerized carrageenans. 

Microwave assisted degradation allowed a reduction in operation times and almost did not change the structure and constitutions of the λ-carrageenan [162], and κ-carrageenan [157] with antiviral properties. However, the special high-pressure equipment needed could be difficult to operate [162]. Operation in closed vessels and in open vessel was reported; additionally, operation in domestic devices could be proposed for acid hydrolysis [157].

During ozonation the depolymerization of polysaccharides causes chemical changes, as well as physicochemical and rheological modifications, since ozonation of κ-carrageenan leads to the formation of carbonyl, carboxyl or double bonds; however, the sulfate groups in k-carrageenans were maintained [177]. 

The ultrasound-assisted depolymerization of κ-carrageenan is simple, suitable for food applications and energy saving, since it is faster than thermal depolymerization at lower temperatures [158]. The susceptibility to ultrasound assisted degradation differs among the carrageenan types, being higher for κ- than for τ-carrageenans [182] and possibly occurs due to the ultrasonically induced breakage of non-covalent bonds in κ-carrageenan molecules [158].

**Table 4:** Examples of technologies used for extraction of carrageenan oligomers.

Depolymerization	Seaweed or Polyssaccharide	Properties	References
Acid hydrolysis	Carrageenan (C)	Mw: κ-, 510–4000; ι-, 110–3300; λ-, 660–5800	[179]
Acid hydrolysis	*Eucheuma cottonii*	DP: κ-, 6–20	[180]
Enzymatic	*Chondrus armatus*, *Kappaphycus alvarezii*, *Tichocarpus crinitus*	Mw: ĸ-, 2.2–4.3	[176,178]
Enzymatic	Carrageenan (C)	Mw: ĸ-, 681–798	[183]
High-Pressure	*Halymenia durvillei*	Mw: λ-, 260–1100	[95]
Irradiation	Carrageenan (C)	Mw: κ-, 8.5–32.1; ι-, 3.1–6.9; λ-, 2.7–6.5	[184]
Microwave assisted	*Solieria chordalis, Chondrus ocellatus*	Mw: λ-, 3–240 Mw: λ-, 650	[152,162]
Ozonization	Carrageenan (C)	Mw: ĸ-, 10–200	[177]
Radical depolymerization	*Halymenia durvillei*	Mw: λ-, 3.3–890	[95]
Subcritical water extraction ionic liquids as catalyst	*Kappaphycus alvarezii*	Mw: ĸ-, 10–60	[185]
Ultrasound assisted	*Kappaphycus alvarezii*, *Eucheuma cottonii*	Mw: ĸ-, 545 Mw: ĸ-, 160–240	[158,182]

C: commercial; Mw: Molecular weight (kDa); DP: degree of polymerization.

Carrageenan can be degraded by gamma irradiation, operating in different systems (solid, gel or solution) at ambient temperature and the molecular weights can be lowered to 8–100 kDa with a narrow distribution, but different yield and susceptibility to degradation occur for the different carrageenan types. Abad [184] reported the use of irradiation with gamma rays at room temperature to depolymerize polysaccharides with enhanced antioxidant properties [186]. Irradiated κ-carrageenan as incorporated as antioxidants in many food systems, but the toxicity of radiolytic products from irradiated κ-carrageenan have to be studied further [184].

Comparative studies have revealed that the method of depolymerization strongly influences the properties of carrageenan oligomers [178]. The chemical depolymerization (free radical or mild acid hydrolysis), produced oligomers with lower Mw (1.2–3.5 kDa) than the enzymatic depolymerization using a recombinant kappa-carrageenase from *Pseudoalteromonas carrageenovora*, yielding 2.2 kDa oligomers from *Chondrus armatus* and *Kappaphycus alvarezii* k-carrageenans and 4.3 kDa oligomers from *Tichocarpus crinitus* κ/β-carrageenans. Low molecular weight derivatives obtained by mild acid hydrolysis showed higher antiviral activity than those obtained by free radical depolymerization, which were more active than those enzymatically prepared. Sun et al. [176] observed that mild acid hydrolysis caused higher saccharide degradation than H_2_O_2_ depolymerization and κ-carrageenase digestion; the original sulfate content was substantially retained and all the hydrolysates had stronger reducing power than the polysaccharide, with H_2_O_2_ hydrolysates being the most potent. After free radical treatment at 40 °C for 4 h, the low-molecular weight oligosaccharides from κ-carrageenan ranged from disaccharide to octasaccharide. The degradation with a κ-carrageenase hydrolyzing the β-1,4 linkages to a series of homologous, even-numbered oligosaccharides (An-G4S)n, yielding 2, 4, 6, 8 and 10 DP, being dominant the tetra- and hexasaccharides [176]. Whereas for H_2_O_2_ treatment, the scavenging ability increased with time as a result from the increment of –COOH groups, the scavenging ability of HCl hydrolysates and enzymatic hydrolysates decreased when the molecular weight decreased. Other combinations have been suggested, i.e., radical depolymerization, and high-pressure homogenization led to several samples of various and controlled molar masses of *Halymenia durvillei* [95].

### 2.2. Protein

The protein content in red algae, higher than in brown and green groups, accounts for 10–50% of the dry weight, being comparable or higher than in some foods [187,188] and the essential aminoacid content, accounting for 25–50% of the total amino acids, is similar as in other protein sources such as casein, ovalbumin and leguminous [3,189,190,191]. The protein contents differ according to the species and seasonal conditions [187,188], being the highest in *Porphyra*, followed by *Palmaria* sp. Nitrogen-to-protein conversion factors of 4.92, lower than for brown and green algae have been proposed [192,193] and algae may contain non-protein nitrogen, resulting in an overestimation of their protein content. Although the digestibility of proteins seems to be limited by the algae non-proteic fraction [187,189] they have been proposed for inclusion in diets of ruminants, hens, rabbit, poultry and pigs [3].

**Figure 4:**
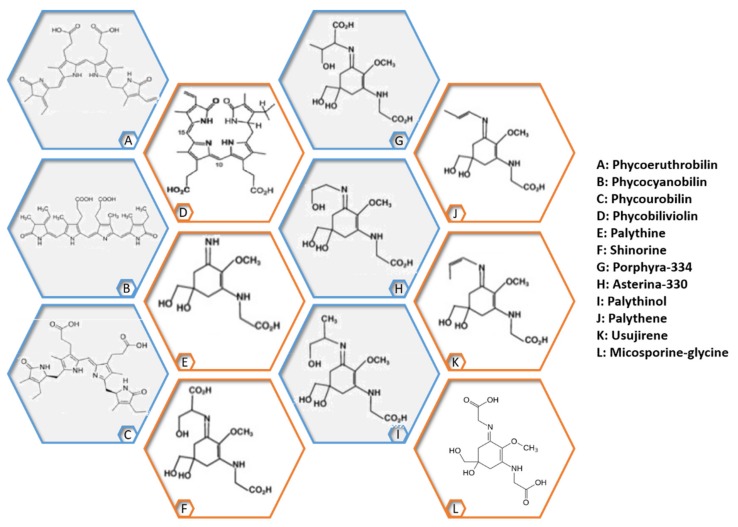
Structure of phycobiliproteins and micosporine-like-aminoacids, adapted from [16,188].

Red algae have a characteristic bright pink color caused by phycobiliproteins. Phycobiliproteins are covalently bound via cysteine amino acids to pigmented phycobilins [3,16]. They are classified into phycoerythrin (red) and phycocyanin (blue). The two types of phycoerythrin (PE) were named after the taxa of the organism form which they were first isolated: R-PE from Rhodophyta and B-PE from Bangiales. Phycocyanins are further subdivided into C-phycocyanin, R-phycocyanin, allophycocyanin or phycoerythrocyanin. Examples of phycobiliproteins found in red seaweeds are shown in Figure 4.

Phycobiliproteins are commercially used in foods, nutraceuticals, cosmetics as a colorant and for their therapeutic value, namely their antimicrobial, antioxidant, anti-inflammatory, neuroprotective, hepatoprotective, immunomodulating and anticarcinogenic properties [16,194,195,196,197,198,199,200,201]. They can improve the efficacy of standard anticancer drugs, lower their side effects [202] and act as photosensitizers for the treatment of tumoral cells [203]. They are also used as fluorescent markers in clinical diagnostics and immunological analysis. 

The storage conditions influence the preservation of phycoerythrin (R-PE) and freezing was reported as the best preservation method [204]. Significant changes in phycoerythrin and phycocyanin were also observed after different culinary treatments [205]. Whereas drying and hydration did not affect the content of phycoerythrin, boiling and steaming caused lowered values.

- Extraction processes: conventional and emerging technologies

Since the extraction of proteins from seaweeds is complicated by the presence of cell wall polysaccharides, the classical procedures are based on the use of buffer, osmotic shock, detergents or the application of alkali treatment, some examples are summarized in Table 5. Different physico-chemical and enzymatic pretreatments have been suggested to enhance the yields, such as repeated freeze-thaw cycles [201,206], or grinding in liquid nitrogen of the freeze-dried seaweeds [207] aided in the release of R-phycoerythrin. Their further purification from the crude extract has been usually addressed through ammonium sulfate precipitation [208] or also by sucrose step-gradient ultracentrifugation [209] followed by purification by gel filtration and by ion exchange chromatography [130,210,211,212].

The use of enzymes degrading the cell wall polysaccharides as an alternative method to improve the extraction and the solubilization of algal proteins [188], since firstly reported by Amano and Noda [213] suggested the use of a mixture of enzymes from the gut of abalone *Haliotis discus* and a commercial one to enhance the extraction of proteins from *Porphyra yezoensis*. Enzyme assisted extraction could improve the physicochemical characteristics, volatile compounds and organoleptic quality of plant proteins producing peptides and amino acids with less salt and carcinogenic compounds than acid hydrolysis [214]. Both the extraction efficiency and the composition of the extracts depended on the seaweed [215], but the influence of the type of enzyme is also determinant on yields, composition and properties. Whereas some cellulases enhanced the protein extraction yields when used alone [190,216], in other studies, polysaccharidases alone or in mixtures caused only a partial digestion of seaweed cell walls and did not improve the yields [217,218] and mixtures of cellulase with carrageenase or agarase were more favorable [201,217]. Proteolytic hydrolysis is usually proposed to obtain bioactive peptides; however, the protease treatments also enhanced the extraction of antioxidants from *Palmaria palmata* compared to carbohydrases and cold water extraction [190].

**Table 5:** Red seaweed protein extraction.

Technologies	Seaweed	Product	Properties	Reference
Accelerated solvent extraction (acetone or methanol)	*Porphyra umbilicalis*	Carbohydrate/Phlorotannin extraction	Antioxidant	[219]
Carbohydrase hydrolysis under high hydrostatic pressure	*Palmaria palmata, Solieria chordalis*	Antioxidant peptides	Antioxidant	[220]
Enzyme hydrolysis with: protease, agarase, carrageenase, xylanase, cellulase	*Gelidium pusillum Chondrus crispus, Gracilaria verrucosa, Palmaria palmata* *Osmundea pinnatifida, Codium tomentosum, Solieria chordalis*	Antioxidant peptides, protein, phycobiliproteins, R-phycoerythrin	Antioxidant, α-glucosidase inhibition anti-inflammatory	[197,201,215,216,221,222,223,224,225,226,227]
Freezing and thawing	*Porphyra haitanensis, Gelidium pusillum*	Phycobiliproteins (R-PE and R-PC)	Antioxidant	[210,228]
Grinding freeze-dried seaweed in liquid nitrogen	*Mastocarpus stellatus*	R-phycoerythrin	Antioxidant	[207]
Homogenization in water or buffer	*Chondrus crispus, Palmaria palmata, Heterosiphonia japonica, Gelidium pusillum*	Phycobiliproteins (R-PE and R-PC)	Antioxidant, antidiabetic, antitumor	[130,189,228,229]
Osmotic shock	*Palmaria palmata, Polysiphonia urceolata*	Bioactive peptides, R-phycoerythrin	Antioxidant, prevention of atherosclerosis	[210,223]
Subcritical water, optionally catalyst	*Hypnea musciformis, Kappaphycus alvarezii*	Protein, antioxidants, emulsifyiers	Antioxidant, emulsifyier	[230,231]
Ultrasound-assisted extraction	*Palmaria palmata, Porphyra umbilicalis*	Bioactive peptides R-PE and R-PC	Antioxidant	[215,219,223,228]
Ultrasound-assisted extraction	*Gelidium pusillum, Porphyra yezoensis*	R-PE, R-PC, taurine	Antioxidant	[228,232]
Ultrasound and enzyme-assisted extraction	*Osmundea pinnatifida, Codium tomentosum*	Protein	Antioxidant, prebiotic effect	[215]

Additionally, it can be useful in combination with other intensification technologies. Le Guillard et al. [233] reported ultrasound-assisted extraction and ultrasound-assisted enzymatic hydrolysis with an enzymatic cocktail for the extraction of R-phycoerythrin from *Grateloupia turuturu*. They recommended the use of 22 °C to avoid R-PE destruction, and 40 °C when the objective was liquefaction. Enzymatic hydrolysis was combined with mechanical methods, namely, ultrasonication [201]. Suwal [220] reported on the use of a non-thermal high hydrostatic pressure (400 MPa, 20 min) processing combined with polysaccharidases to improve the extraction of proteins, polyphenols and polysaccharides from *Palmaria palmata* and *Solieria chordalis*; the effect of this technique being dependent on the seaweed species and the enzyme used. Mittal et al. [228] compared different pre-treatments for extraction of phycobiliproteins from *Gelidium pusillum* and observed a synergistic effect of ultrasonication when employed in combination with other conventional extraction methods, although ultrasonication alone was not efficient. However, Harrysson et al. [219] observed that the pH-shift protein extraction provided the highest protein yields and concentration in the extracts from *Porphyra umbilicalis*, compared to sonication. Fitzgerald et al. [223] used a papain digestion of crude *Palmaria palmate* protein obtained by osmotic shock and ultrasound assisted extraction, with the aim of obtaining bioactive peptides for the prevention of atherosclerosis and the hydrolysate was nontoxic.

Gereniu et al. [230] extracted protein from *Kappaphycus alvarezii* processed by pressurized hot water extraction. Whereas the hydrolysis efficiency increased from 150 °C to 270 °C, and decreased at 300 °C due to decomposition and protein denaturation, the highest foaming properties were attained at 150 °C, whereas the best emulsifying properties were found at 300 °C. Pangestuti et al. [231] proposed the hydrolysis of *Hypnea musciformis* using subcritical water extraction (120–270 °C) to obtain antioxidant and functional material. They found increased protein and sugar content at 120–150 °C, more marked at higher temperatures (180–210 °C), showing the highest antioxidant activity and thermostable emulsifying properties, which could be related to the increased solubility of protein, to the hydrolysis of oligosaccharides and the degradation of monosaccharides.

Wang et al. [232] reported on the use of ultrasound-assisted extraction during the purification of taurine from *Porphyra yezoensis*. This sulfur-containing amino acid can enhance seafood profile flavour. Homotaurine, an aminosulfonate compound present in different species, has shown in vitro and in vivo neuroprotective effect and could be a promising drug for both prevention of Alzheimer’s disease [234]. Operating at 40 °C and 300 W, the ultrasonic process lowered the extraction time by nine compared to the conventional extraction. 

### 2.3. Lipids and Fatty acids

In red seaweed, lipids and fatty acids are present in low amounts, generally 1–5% of the dry weight [235,236]; however, they contain significantly higher levels or polyunsaturated fatty acids than vegetables and have been proposed as a chemotaxonomic tool to differentiate macroalgae [237]. Macroalgae also contain various other lipids and lipid like compounds such as sterols, phospholipids and glycolipids, but red seaweeds have a high ω-3 fatty acids content, being a rich source of α-linolenic acid (ALA) [18:3(ω3)], AA, eicosapentaenoic acid (EPA) [20:5(n-3)], and docosahexaenoic acid (DHA) [22:6(ω3)]), and most species showed a nutritionally beneficial ω6/ω3 ratio [3,238,239] (Table 6). Some macroalgae present a low ω6/ω3 ratio, the ω3 polysunsaturated fatty acids (PUFAs) cannot be synthesized by humans and are thus obtained only through dietary sources. Their therapeutic, especially eicosapentaenoic acid (EPA), has been shown in the reduction of blood cholesterol, and in the protection against cardiovascular and coronary heart diseases [240], and they have anti-inflammatory, anti-thrombotic and anti-arrhythmic properties [241]. 

Total fatty acid concentrations vary among species, accounting for 1–8 in % of dry weight, showing significant differences in the fatty acid profiles [242], which can also be depending on the storage conditions (time and temperature) and the solvent also influences the yields and composition of the lipid extracts [242].

Kumari et al. [237] compiled the total lipid content and fatty acid distribution of different seaweeds and suggested that the variations observed between different species of the same genus was more likely to be due to the inter-specific/intra-generic variations rather than to geographical and environmental conditions as apparent from the minor variations found with the environmental parameters for the studied collection sites.

- Extraction processes: conventional and emerging technologies

The growing interest in PUFA-rich lipids from seaweeds for incorporation into foods has led to an increasing demand for novel extraction techniques with food grade solvents providing high extraction yields. Supercritical CO_2_ extraction of bioactives (neutral lipids and antioxidants) from microalgae and seaweeds [243] is performed in a non-oxidizing atmosphere, which can prevent degradation. Drying and crushing are required stages despite the high energy consumption of the first stage. Chen and Chou [227] reported similar fatty acid profiles of different red seaweeds extracted by supercritical fluids extraction method; however, Cheung [244] observed increased proportions of *Hypnea charoides* PUFAs with operation pressure. The total fatty acid content and the EPA content in the extract produced by pH-shift was slightly reduced compared to that in the crude seaweed from *Porphyra umbilicalis* [219]. 

Patra et al. [245] reported the use of microwave assisted hydrodistillation to extract the volatile oil from *Porphyra tenera*, which showed radical scavenging properties comparable with BHT and α-tocopherol. Kumari et al. [246] reported on the application of sonication and buffer individually on the lipid extraction from *Gracilaria corticata* with analytical purposes. Table 6 shows the extraction yields and the PUFA ratio for different red seaweed genus obtained with conventional and alternative extraction technologies.

**Table 6:** Total lipid (TL) content, polysunsaturated fatty acids PUFA ratio and distribution in red seaweed extracts.

Seaweed Genus	Extraction	TL (mg/g fr. wt.)	PUFA/SFA	ω6/ω3	Reference
*Acanthophora*	CHF/M/PB	6.8–10.4	0.79–0.94	0.9–1.8	[237]
*Asparagopsis*	CSE (H)	3.0	0.06	0.62	[238]
*Bangia*	SFE	13.3 dw	2.8	2.22	[240]
*Bornetia*	CSE (H)	5.3	0.76	0.29	[238]
*Botryocladia*	CHF/M/PB	2.3–5.2	0.49–0.54	1.7–3.6	[237]
*Coelarthrum*	CHF/M/PB	7.7	0.67	5.7	[237]
*Delisea*	CSE (Et; DCM:M)	2.2	1.35	0.4	[242]
*Galaxaura*	SFE	19.8 dw	0.98	0.71	[240]
*Gastroclonium*	CHF/M/PB	4.3	0.59	5.1	[237]
*Gelidiopsis*	CHF/M/PB	5.5	0.84	0.8	[237]
*Gelidiella*	CHF/M/PB	6.7	0.98	0.6	[237]
*Gracilaria*	CHF/M/PB	2.9–9.7	0.15–2.13	0.6–1.9	[237]
*Grateloupia*	CHF/M/PB; SFE	5.0–6.4, 13.6 dw	0.74–1.4	0.5–1.9	[237,240]
*Griffithsia*	CHF/M/PB	4.2			[237]
*Halymenia*	CHF/M/PB; SFE	10–18.8 dw	1.37–1.8	1.7–5	[237,240]
*Helmintocladia*	SFE	19.7 dw	1.05	1	[240]
*Hypnea*	SCF: 50 °C, 37.9 MPa	5.8–7.8	0.31–0.43	0.8–16	[237,243]
*Jania*	CSE (H)	2	0.79	0.60	[238]
*Jania*	CHF/M/PB	12.2	0.32	2.9	[237]
*Laurencia*	CSE (Et; DCM:M)	5.4–16.0	0.41–1.08	0.4–1.7	[237,242]
*Liagora*	SFE	17.6–21.5 dw	0.94–1.43	0.42	[240]
*Peyssonelia*	CSE (H)	4.8	1.33	1.9	[238]
*Porphyra*	MAHD: 40 W, water	11.2–12.4 dw	2.4–2.5	1.2–9.1	[240,245]
*Pterocladiella*	CSE (H)	5.5	0.51	0.9	[238]
*Pyropia*	CHF/M/PB	7.0–7.7	1.23–1.76	0.7–1.4	[237]
*Rhodymenia*	CHF/M/PB	7.1	0.87	88.2	[237]
*Sarconema*	CHF/M/PB	4.3–9.8	0.27–1.04	2.4–2.5	[237]
*Solieria*	CHF/M/PB	9.0	0.35	0.8	[237]
*Cryptonemia*	CHF/M/PB	11.3	0.86–1.28	0.9–18.8	[237]
*Odonthalia*	CHF/M/PB	11.4	0.72	0.6	[237]
*Polysiphonia*	CHF/M/PB	9.6	0.53	1.1	[237]
*Scinaia*	CHF/M/PB	5.2–17 dw	0.23–1.86	1.1–5.3	[237,240]
*Palmaria*	CSE (Et; DCM:M)	14–46 dw	0.49–1.1	0.21–0.41	[241,247]
*Vertebrata*	CSE (Et; DCM:M)	13–18 dw	0.79	0.4	[247]

CSE: Conventional solvent extraction; CHF/M/PB: chloroform–methanol–phosphate buffer; Et: ether extraction; DCM:M: dichloromethane/methanol; H: hexane; MAHD: Microwave assisted hydrodistillation; SFE: Supercritical fluid extraction.

### 2.4. Extractives

The solvent influences the composition and activity as well as the mechanism of action of extracts [248]. Many studies aimed at the solvent extraction of polyphenols, flavonoids and carotenoids [249]. Organic solvents, such as ethanol, methanol, acetone or their mixtures such as chloroform:methanol, have been used for the extraction of antioxidant components, some illustrative examples are shown in Table 7. The choice of extracting solvents with different polarities can have a significant effect due to the different nature of compounds present in the seaweeds and also species–species differences. Intensification with ultrasound was suggested to enhance the solvent extraction process [250].

Supercritical fluid extraction with pure carbon dioxide can be favorable for the extraction of apolar compounds [13], the addition of a small amount of polar modifiers may increase the affinity of this solvent for relatively polar compounds. Zheng et al. [251] obtained extracts, mainly composed by sesquiterpenes, ketones, fatty acids, phenols and sterols from *Gloiopeltis tenax* by supercritical carbon dioxide extraction with ethanol as modifier, and reported remarkable antioxidant and antimicrobial activity. Ospina et al. [252] reported the extraction of *Gracilaria mammillaris* extracts compounds with antioxidant activity using supercritical CO_2_ modified with ethanol. 

When the simultaneous extraction of different components is addressed, the selection of the enzyme activities could be relevant, i.e., for the extraction of phenolics from *P. palmate*, protease provided higher contents than water extract, whereas some carbohydrases showed lower contents, an effect ascribed to their ability of proteases to liberate LMW peptides and amino acids by proteases, which could also enhance the scavenging activities of the extracts [190]. Combination of enzyme digestion with cellulase and hemicellulose, which disrupted or weakened the structural integrity of the seaweed cell wall and high hydrostatic pressure (HHP) increased the accessibility of enzymes, accelerating the release of intracellular polyphenols from *P. palmata*, and from *S. chordalis* [220]. In some cases, organic solvent extraction was more efficient than emerging techniques, i.e., for phenolics from *Osmundea pinnatifida*, and *Codium tomentosum* and was more efficient than hot water extraction or than enzyme or ultrasound assisted extraction [215]; however, the benefits of using greener solvents have to be considered.

**Table 7:** Examples of extraction of bioactives from red seaweeds.

Solvent	Seaweed	Activity	Reference
Ethanol (70–80%), methanol (80%), Acetone, ethyl acetate, chloroform:methanol (2:1) (80%), dimethyl sulfoxide (80%)	*Gracilaria changii, Gelidium amansii, Kappaphycus alvarezii, Osmundea pinnatifida, Codium tomentosum, Gracilaria lemaneiformis*	Antioxidant, glucose uptake regulation, anti-diabetic, neuroprotective, gastroprotective	[215,248,249,253,254]
Enzyme (proteases, carbohydrases) assisted	*Parmaria palmate*	Antioxidant	[190]
Phosphate buffer	*G. amansii*	Antitumoral	[248]
Ultrasound-assisted	*Laurencia obtusa*	Antioxidant	[250]
Supercritical CO_2_	*Gloiopeltis tenax, Gracilaria mammillaris*	Antioxidant, antimicrobial	[251,252]
Enzyme and high hydrostatic pressure	*Palmaria palmate, Solieria chordalis*	Antioxidant	[220]

Mycosporine-like amino acids are low-molecular-weight, water-soluble components with antioxidant and photoprotective properties found in red seaweeds. Since they have been reported as the strongest UVA-absorbing compounds in nature, they have been proposed as photoprotective materials for skin care products. The antioxidant and antiproliferative activities and mycosporine-like amino acid depended on locations varying in UV-exposure [255], with higher values summer and in shallow waters than in deeper waters [255,256]. Conventional extraction with organic solvents has been reported, i.e., methanol [255], but the ultrasound assistance was also proposed to obtain UV-absorbing compounds [257].

### 2.5. Minerals

Seaweeds are particularly rich in minerals and trace elements, showing ash contents, in the range 20–40% *w*/*w*, and could be a good source of K, Ca, Fe, Mg and other trace elements essential for human nutrition [246,258,259]. Seaweeds concentrate minerals due to their capacity to retain inorganic marine substances from seawater based on the characteristics of their cell surface polysaccharides [23], and contain 10–20 times the minerals of land plants. The Na/K ratios were below 1.5 and can be proposed for low sodium diets, since diets with a high Na/K ratio have been related to the incidence of hypertension. 

Jaballi et al. [260] reported the ability of a mineral and antioxidant-rich extract from *Chondrus canaliculatus* to improve the toxicity caused by a fungicide in adult rat, being effective against hematotoxicity, genotoxicity and oxidative stress in the blood and bone and maintained osteomineral metabolism and bone histo-architecture. 

## 3. Combined Extraction

Most of the proposed extraction processes are not selective and apart from the target compound, others can also be obtained. This could be illustrated with some examples. Extraction by enzymatic hydrolysis of R-phycoerythrin from *Gracilaria verrucosa* causes the release of a small amount of polysaccharides, which could be recovered in the coproduct [225]. Some intensification technologies also favor the simultaneous extraction of different components. During protein extraction, the mineral content in the extracts could be enhanced using accelerated solvent extraction produced extracts compared to that of conventional extracts, whereas the pH-shift-produced extracts had lower ash content than the whole biomass. Although the co-extraction of other compounds different from the target ones could difficult and make more expensive the purification stages, the presence of other high-value food components could confer additional value and synergistic functional and biological properties to the final product. This could occur, as fatty acids together with proteins could be of interest for producing multi-functional protein extracts [219]. 

Some authors proposed the use of more than one fraction, such as the sequential extraction of R-phycoerythrin and agar from *Gracilaria verrucosa* [226], Yuan et al. [254] proposed an initial extraction of the pigments for decolorization of *Gracilaria lemaneiformis* before agar production, allowing also to recover the removed fractions as natural antioxidants. Niu et al. [261] observed that after water extraction of proteins from *Gracilaria lemaneiformis* and further purification of R-phycoerythrin the remaining biomass was used for agar extraction. The yield of agar and its properties showed no significant difference from those obtained from the direct agar extraction from the dried algae. However, the R-phycoerythin recovery and purity were lower than when it was extracted from fresh algae. The most frequent approach consists on the valorization of the waste fractions after phycocolloid extraction as a source of protein. Cian et al. [262,263] reported the use of *Porphyra columbina* wastes to obtain proteins that after proteolystic digestion to produce fractions with immunosuppressive, antihypertensive and antioxidant actions.

Particularly interesting is the integral utilization of the raw material, following a biorefinery approach. In order to make use of all seaweed components requires a rational processing of the whole material, and the algal processing by-products according to the biorefinery concept to allow a complete utilization of biomass [19,264,265]. This alternative processing approach would provide different products and applications, favoring the economics of a process that would not rely exclusively on one product and could be adapted to the demand and needs of different sectors. 

**Figure 5:**
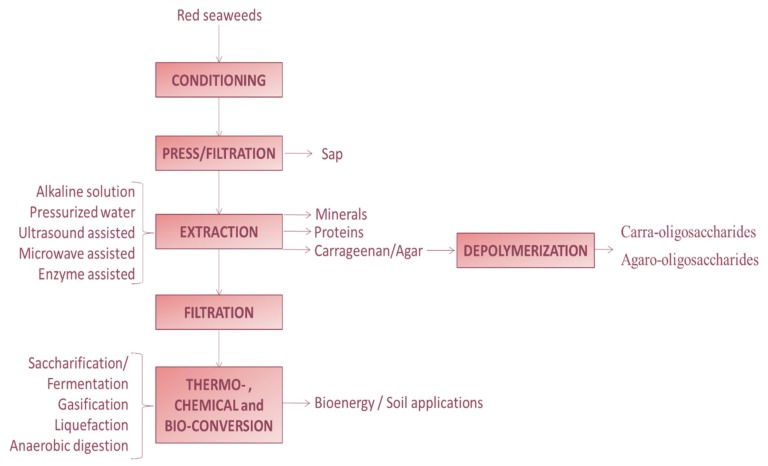
Simplified flow diagram of a red seaweed biorefinery.

Figure 5 represents a general flow diagram for a multistage multipurpose biorefinery processing of red seaweeds. The suggested scheme is based on the initial production of food or feed products, with the final ones being destined to energetic and soil applications. It is desirable that biorefineries are designed in a flexible way allowing the possibility of processing different seaweeds, obtaining different products including those of high volume/low quality and those of high quality/low volume ones. If possible, it is also recommended to integrate food and non-food sectors. In the extraction stages, the utilization of more efficient greener technologies is recommended to enhance the yields and productivities, keeping the products quality and lowering energetic and operation costs. 

Different authors proposed the utilization of agarophytic biomass for biorefinery including the energetic uses [266,267,268,269,270] and for the production of chemicals, such as 5-hydroxymethyl furfural, levulinic acid and formic acid from *K. alvarezii* [271]. However, in the present review, the valorization of bioactives is emphasized, and this type of seaweed is highly interesting, since they contain a high proportion of proteins, some being colorants (R-phycoerythrin, R-phycocyanin), fatty acids and minerals. Therefore, biotechnological, nutraceutical and pharmaceutical applications have been highlighted.

Even when the ethanol production was also considered in their approach, Baghel et al. [264], have designed a complete valorization of *Gracilaria corticata* bioactives, including phycobiliproteins, lipids and agar. The solid residue after phycocolloid extraction is still a good source of bioactives and has been explored in a number of studies. Based on the ingent amounts generated during industrial processing, their valorization would also report environmental benefits. Cian et al. [263] used this waste from *Porphyra columbina* to obtain low molecular weight peptides with angiotensin-converting-enzyme (ACE) inhibitory action, as well as antioxidant properties, which could also be due to some phenolic compounds. Laohakunjit [214] proposed the hydrolysis of *Gracilaria fisheri* residue after agar extraction and the protein hydrolysate was used to obtain free amino acids and odorant compounds valuable as an umami conferring tasting product.

Despite the fact that seaweed biorefineries have started to develop later than terrestrial ones, they offer environmental and economic advantages and show higher potential as a source of nutrients, hydrocolloids, pigments, bioactives and energy, and, based on their complex and exclusive composition, red seaweeds are particularly interesting for their cascading valorization in food, cosmetic and therapeutic applications. 

## 4. Conclusions

The integral utilization of the valuable components from red seaweeds is a technologically feasible approach with environmental and economic advantages. Apart from gelling biopolymers, a number of bioactive compounds with nutritional, functional or biological features can be recovered from red macroalgae using conventional and greener technologies. The challenge is the sequential extraction of these components using emerging technologies for the integral valorization of this type of macroalgae. This opens new attractive alternatives to fulfill the growing market’s demand for natural bioactive compounds of interest in the food, cosmetic, personal care, biomedical or pharmaceutical field.

## Figures and Tables

**Table 2:** Examples of techniques for depolymerization of agar.

Depolymerization Technique	Seaweed or Polysaccharide	Activity	Reference
Acid (HCl, citric acid, and cationic exchange resin (solid acid))	Agar (C)	Antioxidant and -glucosidase inhibition	[77]
Enzymatic	Agarose (C) *Gracilaria cornea* *Gracilaria lemaneiformis*	Functional, antioxidant, skin whitening	[76,83,84,94]
Free-radical induced	*Halymenia durvillei*	ND	[95]
High-pressure homogenization	*Halymenia durvillei*	ND	[95]
Microwave assisted	*Pyropia yezoensis*	Antioxidant	[93]
Ultrasound assisted	*Porphyra yezoensis Gracilaria birdiae*	Anticoagulant, antioxidant	[75,96]

C: commercial; ND: not determined.

**Table 3:** Carrageenan yields and extraction procedures.

Process ^1^	Seaweed	Properties	Reference
^1^ P: 6% KOH, 80 °C, 3 h E: Water, 90–105 °C, 1.5 h, ethanol precipitation	*Hypnea musciformis*, *Kappaphycus alvarezii*, *Solieria chordalis*	Y: 19–27; GS: (4–6.5) × 10^3^; Tg: 32–36, 70–74	[24,152]
P: - E: Water, room temperature, 24 h, ethanol precipitation	*Mastocarpus stellatus*	Y: 15–30 BP: antioxidant, anti-coagulant activities	[17]
^1^ P1,2: 3% KOH, 90 °C, 4 h E1: Water, room temp, 12 h, ethanol precipitation E2: Ultrasound assisted extraction, 15–30 min, 400–500 W, ethanol precipitation	*Kappaphycus alvarezii*, *Euchema denticulatum*, *Hypnea musciformis*	E1: Y: 30–40 E2: Y: 32–49, higher yield with shorter times BP: No differences in antioxidant features	[7,153]
P1: 3% KOH, 85 °C, 3.5 h E1: Water, 85 °C, 12 h, ethanol precipitation P2:- E2: Microwave assisted closed vessels, 85–105 °C, 10–20 min, ethanol precipitation	*Hypnea musciformis*, *Solieria chordalis*	E1: Y: 20–40 E2: Y: 15–25; higher desulfation degree; BP: antiviral	[152,154]
P, E: Alkali extraction, ethanol precipitation	*Chondracanthus acicularis*, *Chondracanthus teedei*, *Gigartina pistillata*, *Chondrus crispus*	Y: 15–45%	[104]

^1^ Optionally, an alkaline pretreatment can be applied; P: pre-treatment conditions; E: extraction conditions; Y: yield (%); GS: gel strength (G’, elastic modulus at 25 °C, Pa); Tg: gelling temperature (° C); BP: biological properties.
